# Closure of Patent Foramen Ovale *versus* Medical Therapy after Cryptogenic Stroke: Meta-Analysis of Five Randomized Controlled Trials with 3440 Patients

**DOI:** 10.21470/1678-9741-2018-0020

**Published:** 2018

**Authors:** Michel Pompeu Barros Oliveira Sá, Luiz de Albuquerque Pereira de Oliveira Neto, Gabriella Caroline Sales do Nascimento, Erik Everton da Silva Vieira, Gabriel Lopes Martins, Karine Coelho Rodrigues, Giulia Cioffi Nascimento, Alexandre Motta de Menezes, Ricardo Felipe de Albuquerque Lins, Frederico Pires Vasconcelos Silva, Ricardo Carvalho Lima

**Affiliations:** 1 Division of Cardiovascular Surgery, Pronto-Socorro Cardiológico de Pernambuco (PROCAPE), Recife, PE, Brazil.; 2 Universidade de Pernambuco (UPE), Recife, PE, Brazil.; 3 Nucleus of Postgraduate and Research in Health Sciences of Faculdade de Ciências Médicas and Instituto de Ciências Biológicas (FCM/ICB), Recife, PE, Brazil.; 4 The CASUAL Investigators - CArdiovascular SUgery Academic League of the Universidade de Pernambuco (UPE), Recife, PE, Brazil.

**Keywords:** Foramen Ovale, Patent, Vascular Closure Devices, Meta-Analysis

## Abstract

**Objective:**

We aimed to determine whether patent foramen ovale closure reduces the risk of stroke, also assessing some safety outcomes.

**Introduction:**

The clinical benefit of closing a patent foramen ovale after a cryptogenic stroke has been an open question for several decades, so that it is necessary to review the current state of published medical data in this regard.

**Methods:**

MEDLINE, EMBASE, CENTRAL/CCTR, SciELO, LI-LACS, Google Scholar and reference lists of relevant articles were searched for randomized controlled trials that reported any of the following outcomes: stroke, death, major bleeding or atrial fibrillation. Five studies fulfilled our eligibility criteria and included 3440 patients (1829 for patent foramen ovale closure and 1611 for medical therapy).

**Results:**

The risk ratio (RR) for stroke in the "device closure" group compared with the "medical therapy" showed a statistically significant difference between the groups, favouring the "device closure" group (RR 0.400; 95% CI 0.183-0.873, *P*=0.021). There was no statistically significant difference between the groups regarding the safety outcomes death and major bleeding, but we observed an increase in the risk of atrial fibrillation in the "device closure group (RR 4.000; 95% CI 2.262-7.092, *P*<0.001). We also observed that the larger the proportion of effective closure, the lower the risk of stroke.

**Conclusion:**

This meta-analysis found that stroke rates are lower with percutaneously implanted device closure than with medical therapy alone, being these rates modulated by the rates of effective closure.

**Table:** 

Abbreviations, acronyms & symbols				
AHA	= American Heart Association		PRISMA	= Preferred Reporting Items for Systematic Reviews and Meta-Analyses
ASA	= American Stroke Association		RCTs	= Randomized controlled trials
CI	= Confidence interval		RR	= Risk ratio
LILACS	= Literatura Latino-Americana em Ciências da Saúde		SciELO	= Scientific Electronic Library Online
MeSH	= Medical Subject Headings		SE	= Standard error
PFO	= Patent foramen ovale			
PICOS	= Population, Intervention, Comparison, Outcome andStudy design			

## INTRODUCTION

### Rationale

The clinical benefit of closing a patent foramen ovale (PFO) after a cryptogenic stroke has been an open question for several decades. Current American Heart Association (AHA)/American Stroke Association (ASA) guidelines do not support the use of PFO closure among patients with PFO and cryptogenic stroke^[1]^. Nevertheless, new randomized controlled trials (RCTs) were published recently, so that controversy still exists over the preferred management strategy for patients with cryptogenic stroke and PFO. Therefore, it is necessary to review the current state of published medical data in this regard.

### Objective

We aimed to determine whether PFO closure reduces the risk of stroke, also assessing some safety outcomes. This analysis was planned in accordance with current guidelines for performing comprehensive systematic reviews and meta-analysis with meta-regression, including the Preferred Reporting Items for Systematic Reviews and Meta-Analyses (PRISMA)^[2]^ guidelines for RCTs. We prespecified our analytical plan and registered the study protocol with PROSPERO, the international prospective register of systematic reviews (CRD42018084583).

## METHODS

### Eligibility Criteria

With the PICOS (Population, Intervention, Comparison, Outcome and Study design) strategy, studies were considered if: 1) the population comprised patients with recent stroke or transient ischemic attack who had a PFO; 2) there was an intervention group of device closure; 3) there was a control group receiving medical therapy; 4) outcomes studied included any of the following: stroke, death, major bleeding, atrial fibrillation; 5) studies were RCTs.

### Information Sources

The following databases were used (until December 2017): MEDLINE; EMBASE; CENTRAL/CCTR (Cochrane Controlled Trials Register); ClinicalTrials.gov; Scientific Electronic Library Online (SciELO); LILACS (Literatura Latino-Americana em Ciências da Saúde); Google Scholar; and reference lists of relevant articles.

### Search

We conducted the search with Medical Subject Headings (MeSH) terms ('Foramen Ovale, Patent' OR 'Patent Oval Foramen ' OR 'Oval Foramen, Patent' OR 'Patent Foramen Ovale') AND ('Stroke' OR 'Cerebrovascular Accident' OR 'Cerebrovascular Accidents' OR 'CVA' OR 'CVAs' OR 'Cerebrovascular Apoplexy' OR 'Apoplexy, Cerebrovascular' OR 'Vascular Accident, Brain' OR 'Brain Vascular Accident ' OR 'Brain Vascular Accidents' OR 'Vascular Accidents, Brain' OR 'Cerebrovascular Stroke' OR 'Cerebrovascular Strokes' OR 'Stroke, Cerebrovascular' OR 'Strokes, Cerebrovascular' OR 'Apoplexy' OR 'Cerebral Stroke' OR 'Cerebral Strokes' OR 'Stroke, Cerebral' OR 'Strokes, Cerebral' OR 'Stroke, Acute' OR 'Acute Stroke' OR 'Acute Strokes' OR 'Strokes, Acute' OR 'Cerebrovascular Accident, Acute' OR 'Acute Cerebrovascular Accident' OR 'Acute Cerebrovascular Accidents' OR 'Cerebrovascular Accidents, Acute').

### Study Selection

The following steps were taken: 1) identification of titles of records through databases searching; 2) removal of duplicates; 3) screening and selection of abstracts; 4) assessment for eligibility through full-text articles; and 5) final inclusion in study. One reviewer followed steps 1 to 3. Two independent reviewers followed step 4 and selected studies. Inclusion or exclusion of studies was decided unanimously. When there was disagreement, a third reviewer made the final decision.

### Data Items

The crude endpoints were stroke, death (any cause), major bleeding and atrial fibrillation.

### Data Collection Process

Two independent reviewers extracted the data. When there was disagreement about data, a third reviewer checked the data and made the final decision. From each study, we extracted patient characteristics, study design, and outcomes.

### Risk of Bias in Individual Studies

Included studies were assessed for the following characteristics: sequence generation (randomization); allocation concealment (selection bias); blinding of participants and personnel (performance bias); blinding of outcome assessors (detection bias); incomplete outcome data addressed (attrition bias) and selective outcome reporting (reporting bias). Considering these characteristics, the papers were classified into A (low risk of bias), B (moderate risk of bias) or C (high risk of bias). Two independent reviewers assessed risk of bias. Agreement between the two reviewers was assessed with kappa statistics for full-text screening and rating of relevance and risk of bias. When there was disagreement about risk of bias, a third reviewer checked the data and made the final decision.

### Summary Measures

The principal summary measures were risk ratio (RR) with 95% CI and *P* values (considered statistically significant when *P*<0.05) for stroke, death, major bleeding and atrial fibrillation. The metaanalysis was completed with the software Comprehensive Meta-Analysis (version 2, Biostat, Inc., Englewood, NJ, USA).

### Synthesis of Results

Forest plots were generated for graphical presentations of clinical outcomes, and we performed the I^2^ test and χ^2^ test for the assessment of heterogeneity across the studies^[3]^. Inter-study heterogeneity was explored using the χ^2^ statistic, but the I^2^-value was calculated to quantify the degree of heterogeneity across the studies that could not be attributable to chance alone. When I^2^ was more than 50%, significant statistical heterogeneity was considered to be present. Each study was summarized by the difference in means or RR, depending on the outcome analyzed. The RR and differences in means were combined across studies using a weighted DerSimonian-Laird random effects model^[4]^.

### Risk of Bias Across Studies

To assess publication bias, a funnel plot was generated for each outcome, statistically assessed by Begg and Mazumdar's test^[5]^ and Egger's test^[6]^.

### Sensitivity Analysis

Sensitivity analyses included the investigation of the influence of a single study on the overall effect - by sequentially removing one study - in order to test the robustness of the main results, so that we could verify whether any study had an excessive influence on the overall results. Moreover, we also analyzed the pool data regarding the outcome "stroke" according to the presence (or absence) of atrial septal aneurysm (hypermobile septum, defined as a septum primum excursion greater than 10 mm).

### Meta-Regression Analysis

Meta-regression analyses were performed to determine whether the effects of the PFO closure were modulated by prespecified factors. Meta-regression graphs describe the effect of aspirin on the outcome (plotted on the y-axis) as a function of a given factor (plotted as a mean or proportion of that factor on the x-axis). Meta-regression coefficients show the estimated increase in log RR per unit increase in the covariate. Since log RR > 0 corresponds to RR > 1 and log RR < 0 corresponds to RR < 1, a negative coefficient would indicate that as a given factor increases, the RR decreases, and vice versa.

The predetermined modulating factors to be examined were: age (mean - years), male gender (%), hypertension (%), smoking (%), large shunt before the interventions, atrial septal aneurysm and effective closure (freedom from large shunt after the interventions).

## RESULTS

### Study Selection

**Fig. 1:**
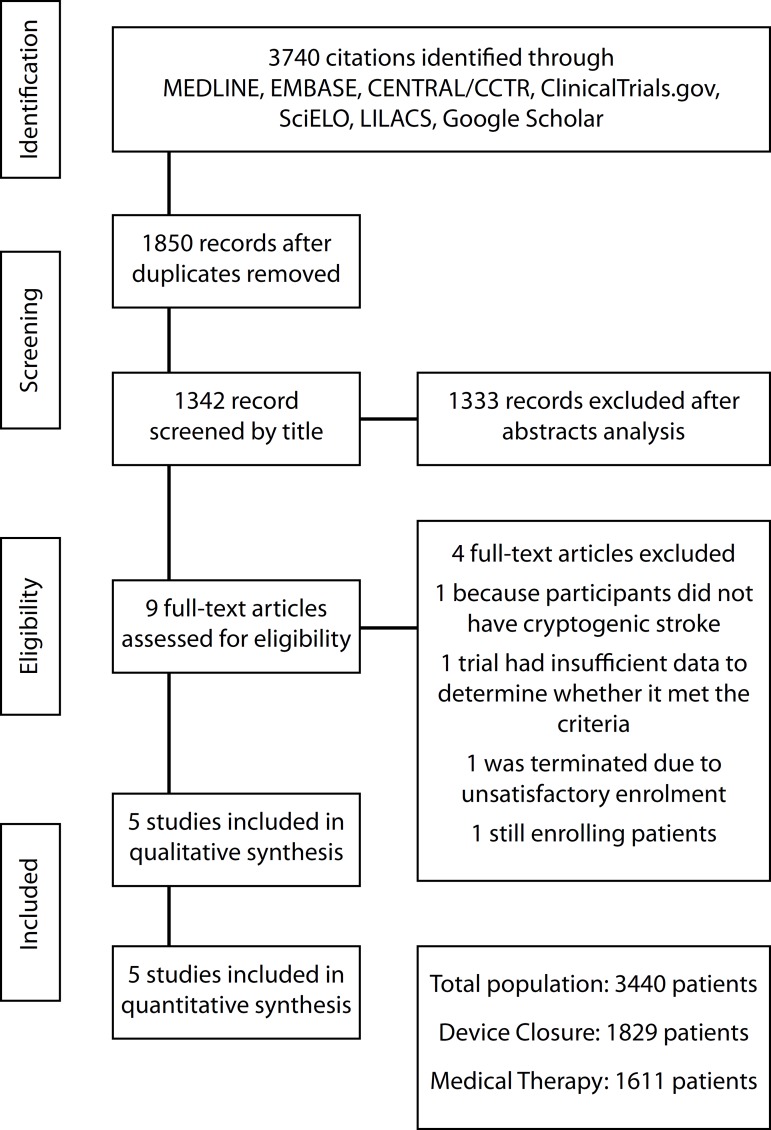
Flow diagram of studies included in data search. CENTRAL/CCTR=Cochrane Central Register of Controlled Trials; LILACS=Latin American and Caribbean Health Sciences Literature; SciELO=Scientific Electronic Library Online

A total of 3,740 citations were identified, of which 9 studies were potentially relevant and retrieved as full-text. Five^[7-11]^ publications fulfilled our eligibility criteria. Interobserver reliability of study relevance was excellent (Kappa = 0.81). Agreement for decisions related to study validity was very good (Kappa = 0.83). The search strategy can be seen in Figure 1.

### Study Characteristics

A total of 3,440 patients (device closure: 1,829 patients; medical therapy: 1,611 patients) were included from studies published from 2012 to 2017. All the trials were multicentric. Most studies consisted of patients whose mean or median age was approximately the fourth decade of life. The medical therapy in the studies was not homogeneous, since different regimens were applied (aspirin, clopidogrel, dipyridamole, combined regimens, etc.). The same goes for the devices used, being the CLOSE trial^[7]^ noteworthy for applying various devices (see Table 1). The overall internal validity was considered "low risk of bias" (Table 2).

**Table 1:** Characteristics of the populations.

	CLOSE (N=473)	REDUCE (N=664)	PC (N=414)	RESPECT (N=980)	CLOSURE (N=909)
% of data in metanalysis	13.7	19.3	12.0	28.4	26.4
Demographic variables					
Age ± SD, years	43.3±10.3	45.1±9.45	44.5±10.2	45.4±9.8	45.5±10.2
Male (%)	58.9	60.1	49.8	54.7	51.8
Medical history variables					
Current smoking (%)	28.9	13.3	23.9	13.3	15.2
Coronary artery disease (%)	NR	NR	1.9	2.9	2.1
Diabetes (%)	2.5	4.2	2.6	7.4	7.8
Hyphercholesterolemia (%)	13.9	NR	27.1	39.5	44.1
Hypertension (%)	10.7	25.6	25.8	31.4	31.0
Migraine (%)	30.6	NR	20.5	38.8	33.6
Prior stroke/TIA (%)	3.6	85	37.4	18.6	12.5
Echocardiographic variables					
Atrial septal aneurysm (%)	32.7	NR	23.7	35.6	35.6
Large shunt (%)	92.8	39.3	21.7	76.1	61.1
Treatment variables					
Randomized to device closure (%)	50.3	66.4	49.3	50.9	49.2
Treated with antiplatelets only (%)	49.6	33.6	80.0	88.0	84.7
Device					
	Amplatzer PFO Occluder or Cribriform; Starflex; CardioSeal; Intrasept PFO; PFOStar; Helex; Premere; PFO occluder OCCLUTECH; PFO occluder GORE (GSO)	EITHER the Helex Septal Occluder device OR the Cardioform Septal Occluder	Amplatzer PFO Occluder (St. Jude Medical)	Amplatzer PFO Occluder (disc occluder)	STARFlex septal closure system (umbrella occluder)

**Table 2:** Analysis of risk of bias: internal validity.

Study	Randomization	Selection bias	Performance bias	Detection bias	Attrition bias	Reporting bias
CLOSE 2017	A	A	B	A	A	A
REDUCE 2017	A	A	B	A	A	A
RESPECT 2013	A	A	A	A	A	A
PC 2013	A	A	A	A	A	A
CLOSURE I 2012	A	A	A	A	A	A

A=risk of bias is low; B=risk of bias is moderate; C=risk of bias is high; D=incomplete reporting

### Synthesis of Results

**Fig. 2:**
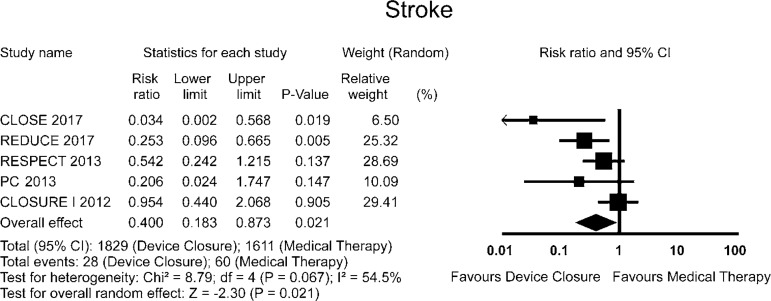
Forest plots of efficacy outcomes.

The RR for stroke in the "device closure" group compared with the "medical therapy" group in each study is reported in Figure 2. There was evidence of moderate heterogeneity of treatment effect among the studies for stroke. The overall RR (95% CI) of stroke showed a statistically significant difference between the groups, favouring the "device closure" group (random effect model: RR 0.400; 95% CI 0.183-0.873, *P*=0.021).

**Fig. 3:**
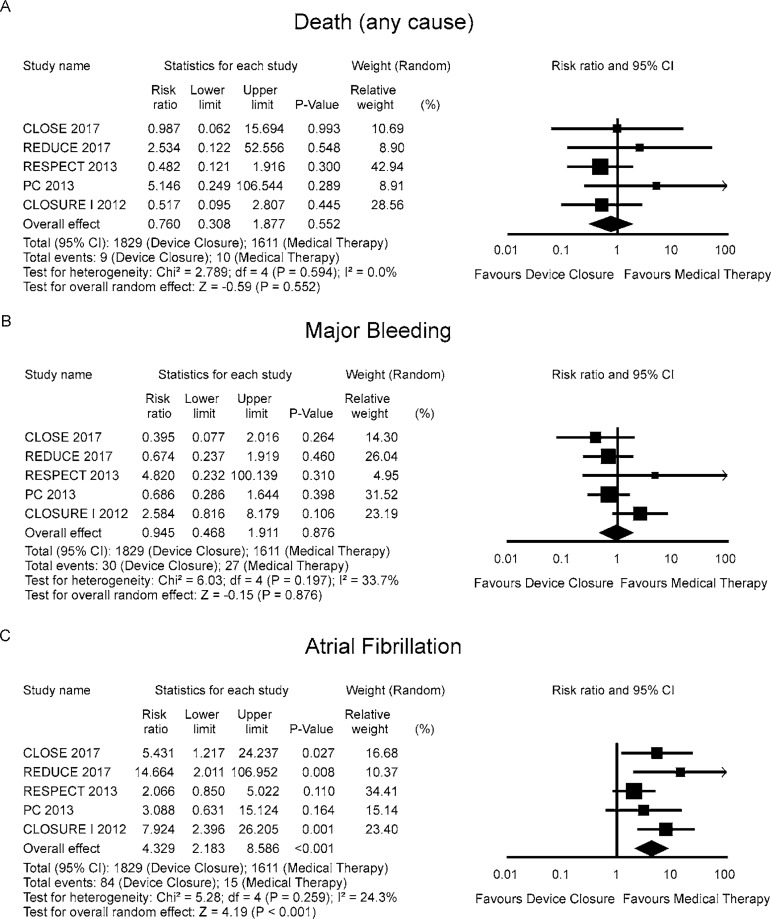
Forest plots of safety outcomes.

The RR for death in the "device closure" group compared with the "medical therapy" group in each study is reported in Figure 3A. There was no evidence of heterogeneity of treatment effect among the studies for death. The overall RR (95% CI) of death showed no statistically significant difference between the groups (random effect model: RR 0.760; 95% CI 0.308-1.877, *P*=0.552).

The RR for major bleeding in the "device closure" group compared with the "medical therapy" group in each study is reported in Figure 3B. There was evidence of mild heterogeneity of treatment effect among the studies for major bleeding. The overall RR (95% CI) of major bleeding showed no statistically significant difference between the groups (random effect model: RR 0.945; 95% CI 0.468-0.873, *P*=1.911).

The RR for atrial fibrillation in the "device closure" group compared with the "medical therapy" group in each study is reported in Figure 3C. There was evidence of mild heterogeneity of treatment effect among the studies for atrial fibrillation. The overall RR (95% CI) of atrial fibrillation showed a statistically significant difference between the groups (random effect model: RR 4.000; 95% CI 2.262-7.092, *P*<0.001).

### Risk of Bias Across Studies

**Fig. 4:**
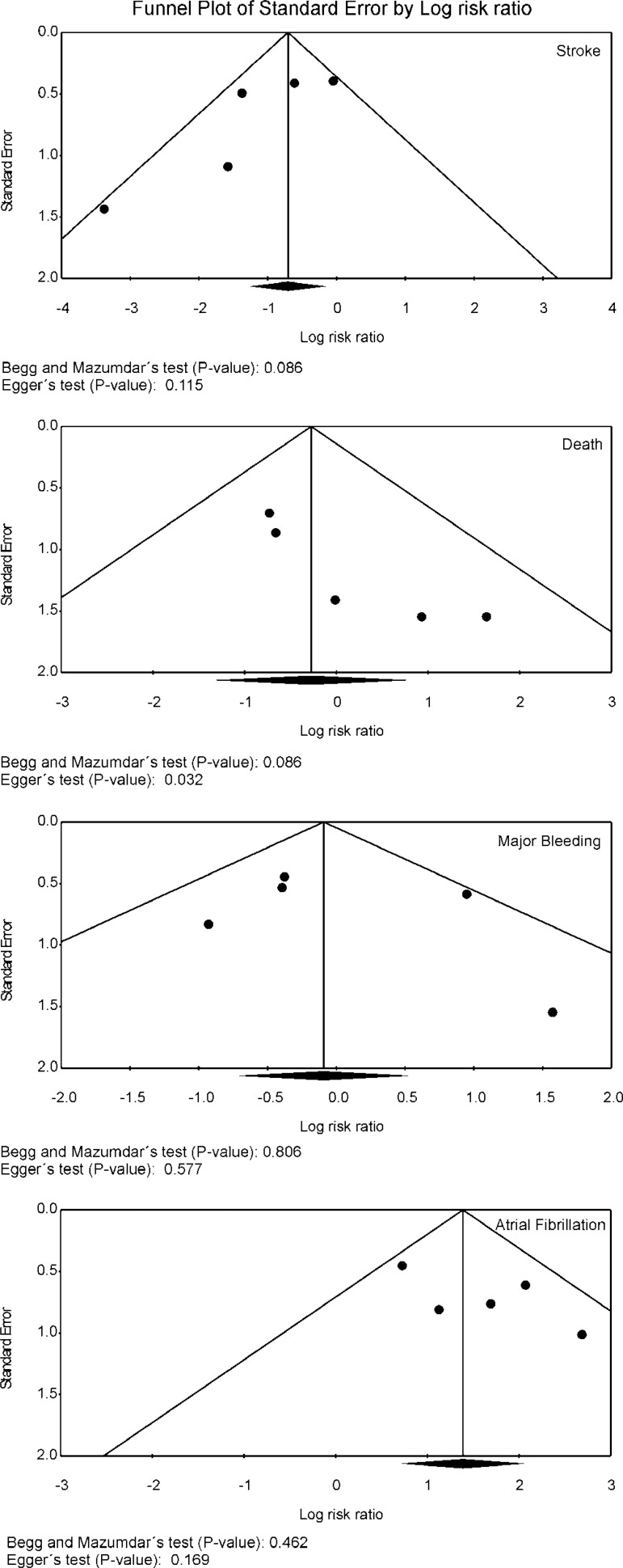
Publication bias analysis of clinical outcomes by funnel plot graphic.

Funnel plot analysis (Figure 4) disclosed no asymmetry around the axis for the outcomes stroke, major bleeding and atrial fibrillation, which means that we have low risk of publication bias related to these outcomes. However, we detected a possibility of publication bias for the outcome death.

### Sensitivity Analysis

**Fig. 5:**
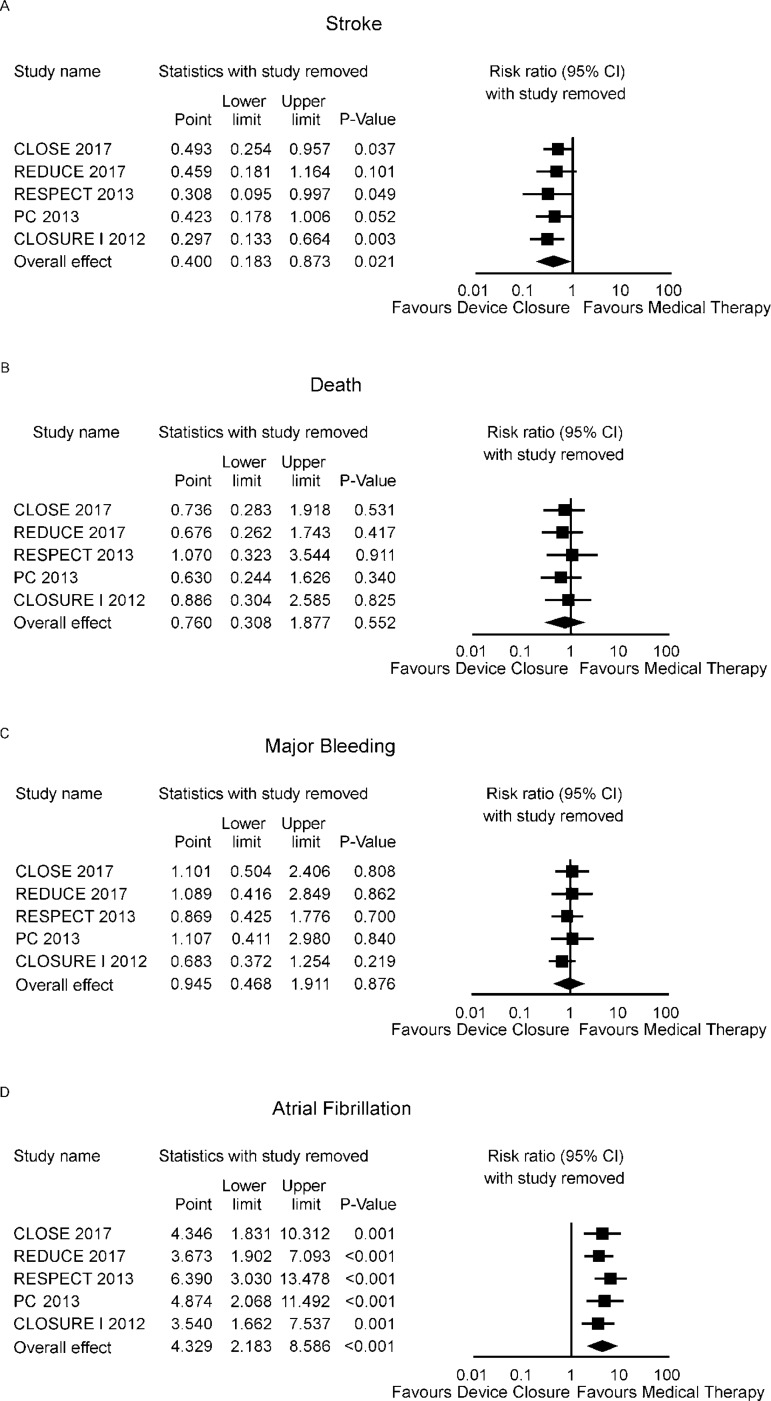
Sensitivity analysis - one study removed.

Sensitivity analyses performed by removing each single study from the meta-analysis to determine the influence of individual data sets to the pooled RR, showed that none of the studies had a particular impact on the results (Figure 5).

**Fig. 6:**
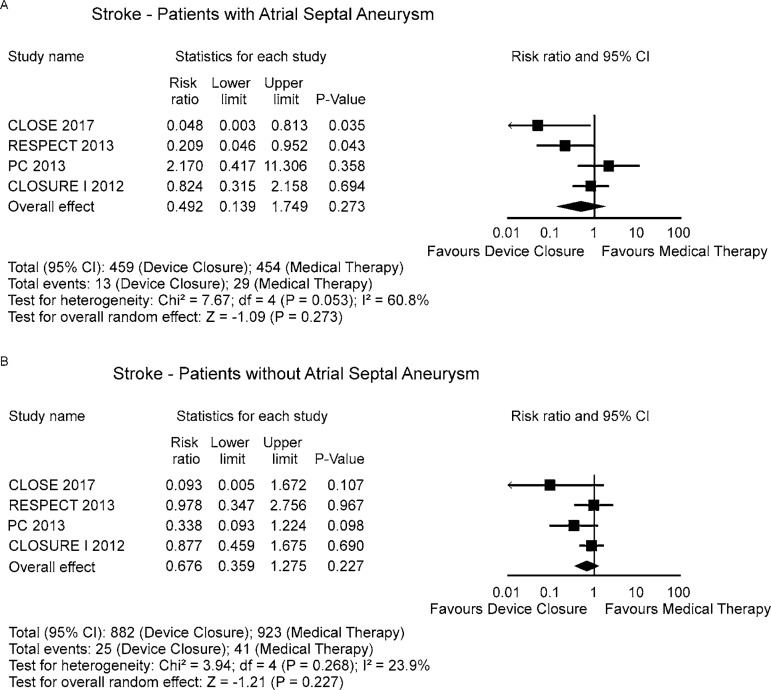
Sensitivity analysis for the presence of an atrial septal aneurysm.

Searching for evidence of a particular impact of the presence of an atrial septal aneurysm on the results, we detected no difference between the groups (Figure 6). Unfortunately, the REDUCE trial^[8]^ was left out of this last analysis because the presence of an atrial septal aneurysm was determined at the time of the PFO closure procedure and, therefore, it was not recorded before trial entry or among the patients in the antiplatelet-only group.

### Meta-Regression Analysis

**Fig. 7:**
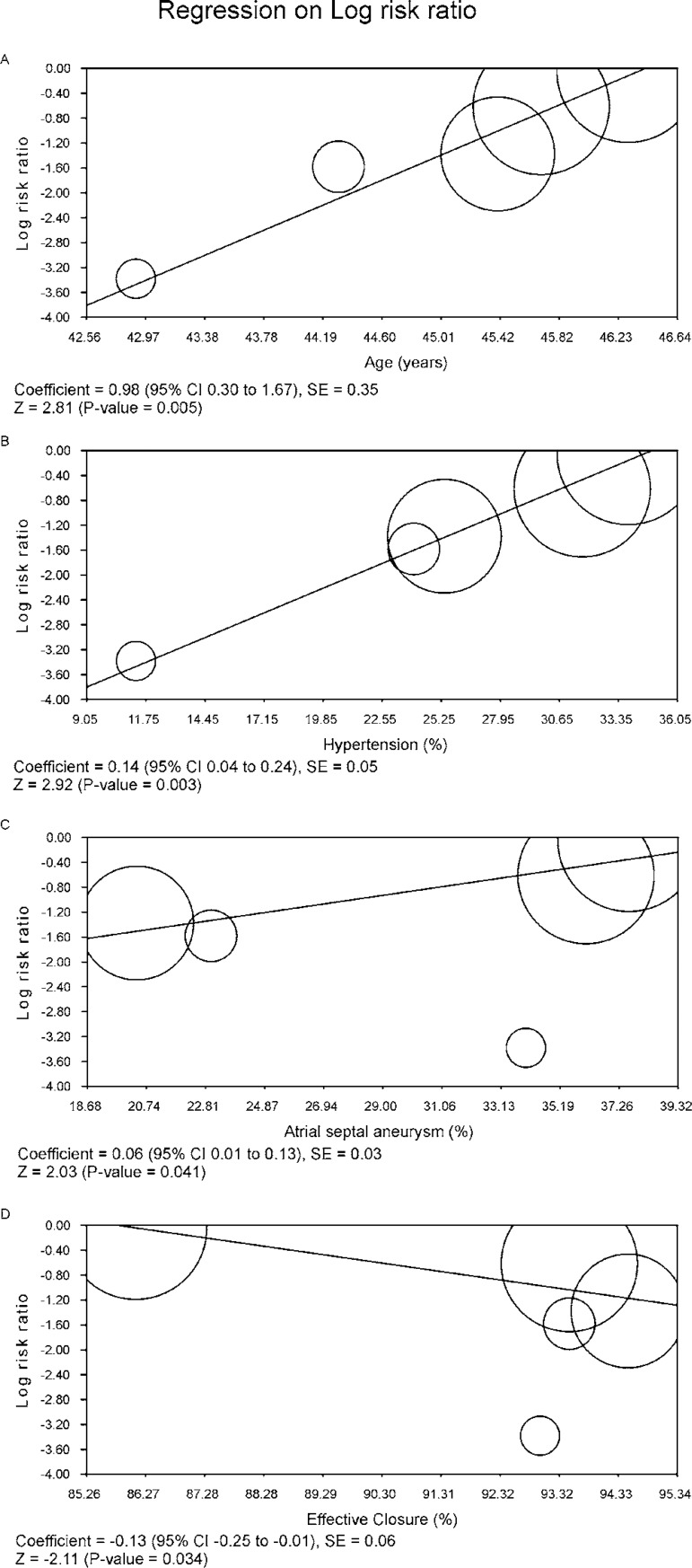
Meta-regression analysis.

Meta-regression coefficients were statistically significant for, age, hypertension, atrial septal aneurysm and effective closure regarding the outcome "stroke". For the variables age, hypertension and atrial septal aneurysm, we observed that the older the patients, the larger the proportion of patients with hypertension and the larger the proportion of patients with atrial septal aneurysm, the higher the risk of stroke (Figures 7A, 7B, 7C). Conversely, the larger the proportion of effective closure, the lower the risk of stroke (Figure 7D).

## DISCUSSION

### Summary of Evidence

To our knowledge, this is the largest meta-analysis of studies performed to date that provides incremental value by demonstrating that patients seem to benefit from device closures in comparison to medical therapy in the reduction of the rate of stroke. On the other hand, there was an increase in the rates of atrial fibrillation. We did not identify the group of patients with an atrial septal aneurysm as a particular group that benefits from the device closure in the sensitivity analysis, although we identified this variable as a risk marker for stroke in the meta-regression. We also observed that the benefit of the device closure in the reduction of the rates of stroke hinges on the rate of effective closure.

### Some Considerations

About 25% of the population has a PFO, but the condition in itself does not increase the risk of ischemic stroke^[12]^. PFO is more prevalent, however, among patients who had a cryptogenic ischemic stroke than in the general population^[13]^. Therefore, we must be careful when selecting patients who would receive some benefit of PFO closure (in term of the risk of stroke).

Kent et al.^[14]^ carried out a patient-level analysis of the CLOSURE I^[11]^, PC^[10]^ and RESPECT^[9]^ trials (before the publication of the CLOSE^[7]^ and REDUCE^[8]^ trials), demonstrating that the device closure was superior to medical therapy, which turned out to be confirmed in our meta-analysis (in terms of the outcome stroke). The improved efficacy in the CLOSE^[7]^ and REDUCE^[8]^ trials might be owing to more strict patient selection. The REDUCE^[8]^ trial had a very strict criteria to exclude patients with other sources of stroke and the CLOSE^[7]^ trial only included those with an atrial septal aneurysm or large shunt.

### The Role of Atrial Septal Aneurysm

Our results do not suggest that this purportedly high-risk anatomical feature is, by itself, very useful at discriminating patients likely to benefit from closure from those unlikely to benefit. Nevertheless, von Klemperer et al.^[15]^, in a survey of current practice in the United Kingdom, identified that around 80% of the 120 respondents (including cardiologists, stroke physicians and neurologists) agreed that an aneurysmal septum was more likely to implicate the PFO in stroke. Only the CLOSE^[7]^ and RESPECT^[8]^ trials showed isolatedly this difference (as we can see in the Figure 6A), but the pooled analysis did not confirm this finding. On the other hand, we might well recognize that there is a correlation between the presence of an atrial septal aneurysm and the risk of stroke (as we can see in the meta-regression - Figure 7C), which led us to the conclusion that this factor is rather a risk marker than a risk factor. Nevertheless, there is no evidence that we should see it as a primary discriminator between those who should have a PFO closed by means of a device.

### The Role of the Effective Closure

The lack of efficacy observed in the CLOSURE I^[11]^ trial has been put down to ineffective PFO closure in the device arm, with 14% demonstrating significant residual right-to-left shunting, whereas, in the other trials, we observed the following rates: 7% (CLOSE^[7]^), 5.5% (REDUCE^[8]^), 6.5% (RESPECT^[9]^) and 6.5% (PC^[10]^). Our meta-regression showed that the more successful the closure, the lower the risk of stroke in the device group (see Figure 7D). Therefore, we must bare in mind that "procedural success", which was defined in the studies as successful implantation with no complications, does not mean "success of PFO closure", which was defined in the studies as minimal or no shunt after the procedure.

### The Problem of Atrial Fibrillation After the Procedure

The rate of new-onset atrial fibrillation was significantly higher in the PFO closure group than in antiplatelet group in our meta-analysis, with most cases detected within 1 month after the procedure - a finding that suggests that the procedure itself induces atrial fibrillation. Indeed, in the closure group, most of the observed cases of atrial fibrillation were periprocedural. The risk of stroke from atrial fibrillation induced by PFO closure has not been determined in the CLOSE^[7]^ trial. In the REDUCE^[8]^ trial, atrial fibrillation was more commonly reported in the PFO closure group, but it was usually transient and the clinical relevance of atrial fibrillation related to closure and overall risk of stroke requires further investigation. In the CLOSURE I^[11]^ trial, a quarter of the strokes in the closure group were ascribed to atrial fibrillation, and in two of these cases, the patients had deviceassociated thrombus on transesophageal echocardiography.

### Future Data to Come Out

At this moment, Song et al.^[16]^ are carrying out the DEFENSE-PFO trial (Device Closure *Versus* Medical Therapy for Cryptogenic Stroke Patients with High-Risk Patent Foramen Ovale - ClinicalTrials.gov Identifier: NCT01550588), which will shed some additional light on this issue by assessing whether percutaneous device closure of PFO is superior to conventional antithrombotic treatment in preventing stroke recurrence in the cryptogenic stroke patients with high-risk of PFO, which was defined as highrisk of recurrence (PFO size ≥ 2 mm or atrial septal aneurysm or hypermobility by transesophageal echocardiography. This study started in 2012 and will be finished in 2020.

### Risk of Bias and Limitations of the Present Study

There are inherent limitations with meta-analyses, including the use of cumulative data from summary estimates. Patient data were gathered from published data, not from individual patient follow-up. Access to individual patient data would have enabled us to conduct further subgroup analysis and propensity analysis to account for differences between the treatment groups. This meta-analysis included only data from randomized studies, which do not reflect the "real world" but, on the other hand, are less limited by publication bias, treatment bias, confounders, and a certain tendency to overestimate treatment effects observed in the observational studies, since patient selection alters outcome and thus make non-randomized studies less robust.

Moreover, besides statistical heterogeneity in some analyses, there is also the issue of the clinical heterogeneity that might have played some role in the pooled results. For instance, in the CLOSE^[7]^ trial, 11 different devices were appplied for PFO closure. In the antiplatelet-only group and the PFO closure group, 410 (86.7%) patients received aspirin, 51 (10.8%) received clopidogrel, 6 (1.3%) received aspirin with extended-release dipyridamole, and 6 (1.3%) received aspirin with clopidogrel. As we can see, not all of patients were 100% equally treated.

## CONCLUSION

This meta-analysis found that stroke rates are lower with percutaneously implanted device closure than with medical therapy alone, being these rates modulated by the rates of effective closure.

## PERSPECTIVES

### What is known?

The results of the firstly published three RCTs (CLOSURE I^[11]^, PC^[10]^ and RESPECT^[9]^) revealed that PFO closure had a statistically significant effect on the composite of stroke, transient ischemic attack, and death in adjusted but not unadjusted analyses, as published in a previous pooled analysis of individual participant data.

### What is New?

After the publication of the two new RCTs (CLOSE^[7]^ and REDUCE^[8]^), the pooled results of our meta-analysis with the five RCTs confirmed that PFO closure reduced the rates of stroke, but also reinforced the problem of atrial fibrillation after the procedure, whose impact remains unknow. This meta-analysis revealed that the more effective the closure, the lower the risk of stroke.

### What is Next?

The publication of the DEFENSE-PFO^[16]^ trial will add important data to those already available. Longer-term followup of completed trials will enhance our understanding of the effectiveness of PFO closure, but studies of various antithrombotic treatment regimens, including those in patients undergoing PFO closure, are necessary to address important knowledge gaps. We still need to know whether all of the devices are beneficial.

**Table:** 

Authors' roles & responsibilities	
MPBOS	Conception and design, analysis and interpretation of data, drafting of the manuscript, revising it critically for important intellectual content; final approval of the version to be published
LAPON	Collection of data, drafting of the manuscript, revising it critically for important intellectual content; final approval of the version to be published
GCSN	Collection of data, drafting of the manuscript, revising it critically for important intellectual content; final approval of the version to be published
EESV	Collection of data, drafting of the manuscript, revising it critically for important intellectual content; final approval of the version to be published
GLM	Collection of data, drafting of the manuscript, revising it critically for important intellectual content; final approval of the version to be published
KCR	Collection of data, drafting of the manuscript, revising it critically for important intellectual content; final approval of the version to be published
GCN	Collection of data, drafting of the manuscript, revising it critically for important intellectual content; final approval of the version to be published
AMM	Revising it critically for important intellectual content; final approval of the version to be published
RFAL	Revising it critically for important intellectual content; final approval of the version to be published
FPVS	Revising it critically for important intellectual contente; final approval of the version to be published
RCL	Revising it critically for important intellectual content; final approval of the version to be published
